# Posterior Capsular Outcomes of Pediatric Cataract Surgery With In-The-Bag Intraocular Lens Implantation

**DOI:** 10.3389/fped.2022.827084

**Published:** 2022-04-08

**Authors:** Yana Fu, Dandan Wang, Xixia Ding, Pingjun Chang, Yinying Zhao, Man Hu, Zhangliang Li, Yun-e Zhao

**Affiliations:** ^1^School of Ophthalmology and Optometry, Eye Hospital, Wenzhou Medical University, Wenzhou, China; ^2^Key Laboratory of Vision Science, Ministry of Health, Wenzhou, China; ^3^National Center for Clinical and Medical Research, Wenzhou, China

**Keywords:** posterior capsular opening area, pediatric cataract surgery, primary in-the-bag IOL implantation, anterior vitrectomy, capsulorhexis diameter

## Abstract

**Aim:**

To investigate the change of posterior capsular outcomes of pediatric cataract surgery with primary in-the-bag intraocular lens (IOL) implantation.

**Methods:**

We conducted a case series of pediatric cataract children who underwent cataract extraction with primary in-the-bag IOL implantation, posterior capsulorhexis or vitrectorhexis, and limited anterior vitrectomy at the Eye Hospital of Wenzhou Medical University between 2016 and 2019. Digital retro-illumination photographs of pediatric eyes were obtained at baseline and 6 months, 12 months, and the last visit postoperatively. Capsular outcomes of the posterior capsular opening area (PCOA) and lens reprolifration area at those time points were compared. Correlations between the PCOA and influential factors, such as age at surgery, axial growth, and follow-up duration, were analyzed. The study was registered at register.clinicaltrials.gov (NCT04803097).

**Results:**

Data of 23 patients (27 eyes) were used in the final analysis. During follow-up, the PCOA enlarged at a rate of 0.29–0.32 mm^2^/month during the first six months postoperatively and 0.05–0.08 mm^2^/month over the next 1–2 years. Six months postoperatively, the PCOA enlargement statistically and positively correlated with the follow-up duration and axial growth. The area of lens reprolifration was 0.46 ± 1.00 mm^2^ at six months postoperatively and then remained stable.

**Conclusion:**

The PCOA enlarged rapidly within the first six months after the pediatric cataract surgery with primary IOL implantation. Six months postoperatively, the enlargement of PCOA was positively correlated with follow-up duration and axial growth. Posterior capsulorhexis or capsulectomy should be performed with a diameter of 3.0 to 4.0 mm for good visual axis transparency and the protection of in-the-bag IOL.

## Introduction

Pediatric cataract surgery is challenging because of the high complications including visual axis opacification (VAO), posterior capsulorhexis opening reclosure, anterior capsule contraction, intraocular lens (IOL) eccentricity, IOL dislocation, zonular dehiscence, secondary glaucoma, and so on (1–4). Some recent studies demonstrated several surgical approaches such as optic capture through the posterior capsulorhexis, and bag-in-the-lens which can avoid anterior vitrectomy could effectively prevent the incidence of VAO (5–7). However, the standard approach of posterior capsulotomy with in-the-bag IOL implantation, posterior capsulorhexis, and anterior vitrectomy remains the most popular, especially in underdeveloped areas (7).

Many ophthalmologists have realized that capsule management during pediatric cataract surgery is one of the critical components determining the final postoperative prognosis for vision (7). Due to the difficulty of its examination in children, few studies have focused on the capsular outcomes of pediatric cataract surgery. Studies by Lin et al. (8) and Tan et al. (9) found that the anterior capsular opening (ACO) contracted, and the posterior capsular opening expanded after pediatric cataract surgery without IOL implantation. However, changes in the capsular outcomes in the pediatric cataract extraction with in-the-bag IOL implantation remain unknown. This study aimed to prospectively investigate the changes of posterior capsular opening after pediatric cataract surgery with primary in-the-bag IOL implantation and to evaluate the ideal sizes of the posterior capsulorhexis during surgery.

## Materials and Methods

### Trial Registration

The study was registered at register.clinicaltrials.gov: NCT04803097.

Children who underwent cataract surgery and primary IOL implantation at the Eye Hospital of Wenzhou Medical University (Hangzhou, China) between 2016 and 2019 were included in the study. The research protocol was approved by the Institutional Review Board and Ethics Committee of Wenzhou Medical University and was conducted according to the tenets of the Declaration of Helsinki. Informed consent was obtained from the parents of all participating children. We excluded patients with ocular trauma, corneal disorders, glaucoma, preoperative lens luxation or subluxation, membranous cataract, persistent hyperplastic primary vitreous, surgical or postoperative complications such as glaucoma or suspect-glaucoma and synechia, pupils that could not be sufficiently dilated, and those who could not complete follow-ups or did not have clear digital photographs. The follow-up period was at least one year.

### Surgery

Surgery was performed by the same experienced surgeon (Z.Y.E.). All surgeries were performed under general anesthesia. A 2.2-mm scleral tunnel incision was created at a 12’o clock position and followed by two corneal incisions (1.0mm). For patients older than three years, we performed lens aspiration after anterior continuous curvilinear capsulorhexis, followed by posterior continuous curvilinear capsulorhexis and limited anterior vitrectomy with a 23-gauge microincision vitrectomy system (Accurus, Alcon Laboratories, Fort Worth, TX, United States). While in patients younger than three years old, anterior and posterior vitrectorhexis was performed instead of manual capsulorhexis. The parameters of the Accurus vitrectomy system were set as follows: bottle height 50–60 mmH_2_O, cutting rate 3000 cpm, flow rate 10 cc/min, vacuum 350 mmHg. The diameter of the anterior capsular opening was about 5.0–5.5 mm, and the posterior capsular opening was about 3.0–4.5 mm. The anterior and posterior vitrectorhexis or capsulorhexis diameters were controlled using a spatula with laser markers (1-mm scale) (9). Then, a posterior chamber IOL was inserted into the capsular bag — 8 eyes with AcrySof SA60AT (Alcon, Fort Worth, TX, United States); 16 eyes with Tecnis ZCB00 (AMO, Santa Ana, CA, United States); and 3 eyes with A1-UV (Eyebright, Beijing, China) (10, 11). The IOL power was selected by SRK-T or Holladay II formulas. The target postoperative refraction was based on the patient’s age according to our team’s previous experience (12).

### Measurements of Outcomes and Follow-Up Protocol

**FIGURE 1:**
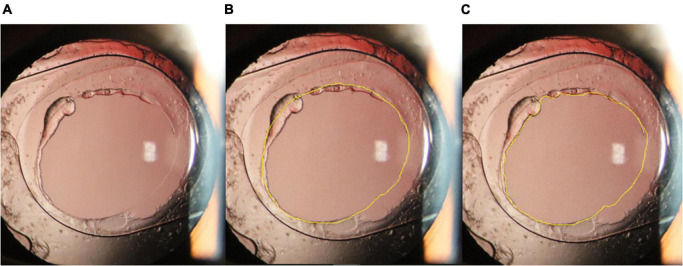
**(A)**: Anterior segment photography of the eye with an intraocular lens one year after surgery. **(B):** Contour of the posterior capsular opening area (yellow line). **(C)**: Contour of the visual axis transparent area (yellow line).

The surgery was video recorded. The photographs of the completed posterior capsulorhexis were obtained before the end of the surgery. Consistency of the anterior chamber was maintained with the balanced salt solution with the same pressure (30 cmH_2_O). All captured images were required to focus on the posterior capsular opening area (PCOA) and visual axis transparent area (VATA) (Figure 1).

Postoperatively, topical eye drops containing 0.3% tobramycin and 0.1% dexamethasone were used four times daily for four weeks, and compound tropicamide eyedrops were used once a day for one month. All patients were examined at 1 week, 2 weeks, 1 month, 3 months, and 6 months postoperatively. After that, patients younger than three years of age were followed up every three months postoperatively for at least one year, and patients three years or older were followed up every six months postoperatively. Visual acuity examinations, refraction, IOP measurements (Icare Finland Oy, Vantaa, Finland), and fundus examinations were performed. Axial length measurements (IOLMaster 500, Carl Zeiss Meditec, Jena, Germany; or Axis Nano ultrasound biometer, Quantel Medical Inc., France) and slit-lamp-adapted anterior segment photography (SLM-8E; Chongqing KangHua S & T Co., Ltd., Beijing, China) were performed at 6, 12, and 18 months postoperatively and then every six months after that. All pediatric patients underwent postoperative pupil dilation with compound tropicamide eye drops three to five times (once every 10 min) until the pupils were fully dilated. Then, slit-lamp-adapted anterior segment digital photographs of each operated eye, including one digital coaxial retro-illumination photograph, were obtained. The patients were sedated through intranasal administration of dexmedetomidine for children who could not cooperate during examinations (13).

### Image Analysis

**FIGURE 2:**
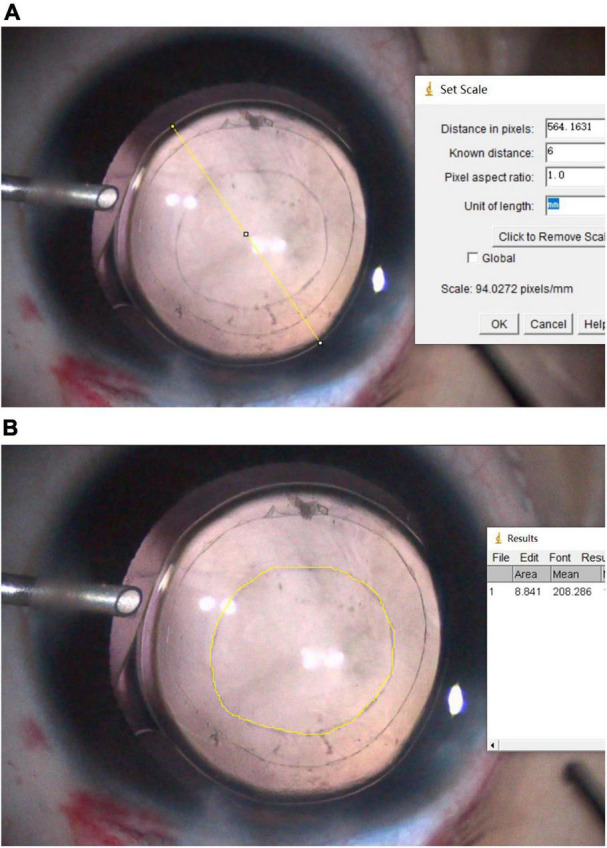
**(A)**: The optic diameter of the intraocular lens (IOL) was marked (yellow line), and the distance (pixelated yellow line) was measured. The original image magnification scale was obtained through the distance in pixels/known distance (the optic diameter of the IOL). **(B)**: The contour of the posterior capsular opening area was marked using a pointer. The original area (mm^2^) was then calculated using the scale included in the Image J software.

Both intraoperative and postoperative pictures were standardized and documented on an analysis computer using ImageJ software (ImageJ 1.51j8; National Institutes of Health, Bethesda, MD, United States), as follows (14): After the images were imported, the optic diameter of the IOL was measured, and the original image magnification scale was obtained. Then, the PCOA and VATA contour was marked using a pointer. The original area was calculated by the software (Figure 2). The lens reproliferation on the visual axis was defined as the difference between PCOA and VATA. Two researchers independently quantified the values and recorded the mean values. The posterior capsular opening diameter (PCOD) and visual axis transparent diameter (VATD) were converted from the PCOA and VATA.


$$PCOD=2\times \sqrt{PCOA/\pi }$$

$$VATD=2\times \sqrt{VATA/\pi }$$

### Statistical Analysis

The SPSS software package (version 19.0; IBM Corp., Armonk, NY, United States) was used for statistical analyses. Data were checked for normal distribution using the Kolmogorov–Smirnov test. A single-sample repeated analysis of variance with Bonferroni correction was used to analyze the parameters observed at all visits. Depending on the data distribution, a Pearson or Spearman correlation analysis was performed to assess the relationship between the PCOA or lens reproliferation and influential factors such as age at surgery. The sample size, which was determined to be 23 subjects (alpha = 0.05; power = 0.90; effect size = 0.717), was calculated for the same primary outcome measure (PCOD) using the PASS software (version 11.0; NCSS, LLC., East Kaysville, UT, United States). A *p-value* of < 0.05 was considered statistically significant.

## Results

Fifty patients (62 eyes) were included in the study. Of these, we excluded 16 patients (20 eyes) due to incomplete follow-up or non-cooperation with the examination; 9 patients (13 eyes) because of poor image quality with insufficient pupil dilation; and 2 patients (2 eyes) because of VAO (1 eye at 6 months and 1 eye at 12 months, postoperatively). Finally, 23 patients (27 eyes) were included in the final analysis of parameters at baseline, 6 months postoperatively, and the last postoperative visit (20.74 ± 7.89 months). The average age of patients at surgery was 3.50 ± 1.80 years (mean ± SD; range, 0.50–6.67 years), including 7 patients (8 eyes) younger than two years of age. There were 12 male and 11 female patients. No severe complications occurred during surgery and the follow-up period. Similarly, there was no noticeable shrinkage or capsular contraction in any of the 27 eyes.

### Results of Image Analysis

Table 1 summarizes the areas and diameters of the posterior capsular opening region and visual axis transparent region at the corresponding time points. These gradually increased from baseline to the last visit. There were significant differences during multiple comparisons between the three time points. The growth rate of PCOA was 0.29 mm^2^/month in the first six months, and 0.08 mm^2^/month from 6 months to the last follow-up (20.74 ± 7.89 months) postoperatively; the corresponding growth rates of PCOD were 0.05 mm/month and 0.01 mm/month, respectively.

**TABLE 1:** Posterior capsular opening/visual axis transparent areas and diameters after surgery.

Parameters (*N* = 27)	V_0_	V_6m_	V_last_	*p-value*			
				V_0_ vs. V_6m_	V_6m_ vs. V_last_	V_0_ vs. V_last_	Total (*p*/F)
PCOD (mm)	3.80 ± 0.53	4.08 ± 0.56	4.25 ± 0.57	< 0.001	<0.001	< 0.001	<0.001/123.455
VATD (mm)	3.80 ± 0.53	4.00 ± 0.58	4.18 ± 0.60	< 0.001	<0.001	< 0.001	<0.001/46.415
PCOA (mm^2^)	11.55 ± 3.29	13.29 ± 3.74	14.43 ± 3.92	< 0.001	<0.001	< 0.001	<0.001/113.779
VATA (mm^2^)	11.55 ± 3.29	12.82 ± 3.85	13.98 ± 4.08	< 0.001	<0.001	< 0.001	<0.001/45.860
LREP (mm^2^)	/	0.46 ± 1.00	0.44 ± 0.90	/		/	0.912/0.012

*PCOD, posterior capsular opening diameter; VATD, visual axis transparent diameter; PCOA, posterior capsular opening area; VATA, visual axis transparent area; LREP, lens reproliferation; V_0_, value (diameter or area) at baseline (during surgery); V_6m_, value (diameter or area) at six months postoperatively; V_last_, value (diameter or area) at the last visit (20.74 ± 7.89 months postoperatively). A single-sample repeated analysis of variance with Bonferroni correction was used.*

Twelve of 23 patients (12 eyes) were available to analyze parameters observed at baseline and 6 months, 12 months, and the last visit (25.64 ± 6.62 months) postoperatively. The corresponding PCOAs were 11.46 ± 3.27 mm^2^, 13.38 ± 3.49 mm^2^, 13.70 ± 3.52 mm^2^, and 14.67 ± 3.77 mm^2^, respectively; the corresponding PCODs were 3.79 ± 0.54 mm, 4.10 ± 0.53 mm, 4.15 ± 0.53 mm, and 4.29 ± 0.56 mm, respectively. After surgery, the growth rates of PCOA were 0.32 mm^2^/month in the first six months, 0.05 mm^2^/month from 6 to 12 months, and 0.07 mm^2^/month from 12 months to the last follow-up (25.64 ± 6.62 months); the corresponding growth rates of PCOD were 0.05 mm/month, 0.01 mm/month, and 0.01 mm/month, respectively.

In the study, there were a total of 8 eyes from 7 patients younger than two years of age (1.25 ± 0.41 years, range: 0.50 ∼ 1.83 years), and 19 eyes from 16 patients older than two years of age (4.44 ± 1.20 years, range: 2.91 ∼ 6.67 years). In the first six months postoperatively, the PCOA growths were 1.80 ± 0.72 mm^2^ in patients < 2 years of age and 1.70 ± 1.23 mm^2^ in patients > 2 years of age (*p* = 0.806, Independent Samples *t*-test). In the period from six months after surgery to the last follow-up, the PCOA growths were 1.08 ± 0.39 mm^2^ in patients < 2 years of age (follow-up time 18.00 ± 8.48 months) and 1.16 ± 0.63 mm^2^ in patients > 2 years of age (follow-up time 21.89 ± 7.55 months, *p* = 0.746, Independent Samples *t*-test).

### Correlation Analysis

Table 2 shows the correlation between the changes in PCOA or lens reproliferation area after surgery and factors such as the growth of axial length, age at the time of surgery, and follow-up duration. The axial lengths at surgery, six months postoperatively, and the last visit (20.74 ± 7.89 months) were 21.61 ± 1.01 mm, 21.62 ± 1.15 mm, and 21.93 ± 1.19 mm, respectively. The change in PCOA (*N* = 27 eyes) in the first six months after surgery did not correlate with axial length growth or age at surgery. However, the difference in PCOA from six months to the last visit postoperatively demonstrated a statistically significant positive correlation with the growth of axial length and the corresponding follow-up duration but not with age at surgery (Table 2). In contrast, the area of lens reproliferation in the first six months after surgery demonstrated a statistically significant negative correlation with age at the time of surgery. However, the area of lens reproliferation at the last visit (20.74 ± 7.89 months postoperatively) did not correlate with age at the surgery or follow-up duration (Table 2).

**TABLE 2:** Correlation between posterior capsular opening area changes or lens reproliferation area and influential factors.

Parameters (N = 27)	Follow-up duration		Age (at surgery)		Growth of axial length at the corresponding time	
	r	*p*	r	*p*	r	*p*
Difference of PCOA (D_last_^&^ – D_0_)	0.219*	0.273*	–0.152^∧^	0.449^∧^	0.041^∧^	0.839^∧^
Difference of PCOA (D_last_^&^ – D_6m_)	0.405*	0.036*	–0.044^∧^	0.829^∧^	0.503^∧^	0.007^∧^
Difference of PCOA (D_6m_– D_0_)	/	/	-0.146^∧^	0.468^∧^	0.012^∧^	0.952^∧^
Difference of Lens Reproliferation (D_last_^&^ – D_0)_	0.032*	0.876*	–0.331*	0.920*	0.033*	0.871*
Difference of Lens Reproliferation (D_6m_ – D_0_)	/	/	–0.762*	0.000*	0.054*	0.787*

PCOA, posterior capsular opening area; D_0_, diameter at baseline (during surgery); D_6m_, diameter at six months postoperatively; D_last_^&^, diameter at the last visit (20.74 ± 7.89 months postoperatively). ^∧^If data conformed to a normal distribution, and then a Pearson correlation analysis was used. *If not, then a Spearman correlation analysis was used.

## Discussion

Capsular opacification and fibrosis are common yet significant complications of pediatric cataract surgery. Although the incidence of VAO has greatly reduced through improved surgical techniques and IOL designs, anterior capsulorhexis shrinkage and posterior capsulorhexis expansion after surgery remain difficult to avoid. Residual LECs in the anterior and equatorial regions of the lens capsule proliferate and undergo fibrous metaplasia (15, 16). Capsular fibrosis along the smooth circular edge of the capsular opening exerts a centripetal force on the anterior capsular opening. When the centripetal force exceeds the centrifugal zonular force on the anterior capsule, it results in anterior capsulorhexis contraction (17). Contrarily, the posterior capsule is generally devoid of LECs. Well polishing of anterior capsule and barrier effect on the epithelium after IOL implantation with a square edge design can reduce cell migration to the posterior capsule, subsequently, reduce fibrous metaplasia (18). Therefore, the centripetal force generated by fibrosis cannot resist the centrifugal force generated by zonular fibers and the supporting force induced by IOL haptics, causing the PCOA to appear enlarged postoperatively. Factors influencing the extent of capsular contraction or expansion include the surgical method, postoperative inflammation, IOL material and design, and the quality of zonular support and capsulorhexis size (8, 17). In addition, there is no clarity on whether factors such as age at the time of surgery and ocular growth have any effect on the capsular bag. Therefore, studying the degree and rate of changes in the posterior or anterior capsular opening area after pediatric surgery is beneficial in clinical treatment.

This is the first study to investigate changes in capsular outcomes after pediatric cataract surgery with primary IOL implantation to the best of our knowledge. We took digital retro-illumination photographs and then analyzed the PCOA through Image J software similar to the previous studies (19). However, this wasn’t easy because the child often did not cooperate with the examination or because the pupil often wasn’t dilated enough. Therefore, many patients were excluded from the experiment for the final data analysis. Even so, our study found some interesting results. During the follow-up of 20.74 ± 7.89 months, the PCOA enlarged rapidly at a rate of 0.29–0.32 mm^2^/month during the first six months postoperatively; after that, it enlarged at a slower rate of 0.05–0.08 mm^2^/month. Six months after the operation, the PCOA enlargement positively correlated with the follow-up duration. Lin et al. (8) first reported capsular outcomes of pediatric cataract surgery without IOL implantation. They found that PCOA in the group with an anterior capsulorhexis diameter of 5.0–6.0 mm, similar to that of patients in our study, enlarged at a rate of 0.28 mm^2^/month during the first six months postoperatively (from 10.37 ± 2.91 mm^2^ at 1 week to 12.07 ± 2.86 mm^2^ at 6 months postoperatively) and at 0.07 mm^2^/month during the next six months postoperatively (12.46 ± 2.95 mm^2^ at 12 months). This trend is similar to our findings, although their baseline was marginally different. Therefore, with or without the IOL implantation, the PCOA expands rapidly in the first six months after surgery due to inflammation or LEC reproliferation and subsequent fibrosis, and then this expansion gradually decreases. As our follow-up period was relatively short, we still need to investigate when the PCOA reaches a steady state.

The size of the anterior capsulorhexis may affect the degree of change of capsular outcomes in pediatric cataracts. Lin et al. (8) reported that the smallest diameter of anterior capsulorhexis yielded the maximum ACO contraction with the minimum PCOA enlargement. In contrast, its largest diameter yielded the minimum ACO contraction with the maximum PCOA enlargement. Initially, we wanted to include similar observations of the ACO; however, our experiments did not succeed. First, the anterior capsulorhexis diameter was designed to be 5.0–5.5 mm, which is the optimal size for age-related cataracts (20–22), with 0.5- to 1.0-mm capsulorhexis edges covering the IOL optic surface. After surgery, the ACO boundary was often challenging to identify due to the interference of the IOL edge. Second, several pupils were not fully dilated to expose the whole ACO boundary. We did not observe any zonular dehiscence or IOL dislocation caused by ACO contraction or PCOA expansion during this study, which may be due to the sufficient intraoperative polishing of the capsule and appropriate anterior capsulorhexis size (23, 24). Currently, there is no definite standard for the ideal size of anterior capsulorhexis for children, which varied from 3.0 to 6.0 mm according to different surgeons (8, 25, 26). Our study revealed that the anterior capsulorhexis diameter of 5.0–5.5 mm was safe and suitable for pediatric cataract surgery with in-the-bag IOL implantation, considering the safety of the capsular bag and the low incidence of VAO.

Data from 12 eyes (baseline, 6 months postoperatively, 12 months postoperatively, and last visit at 25.64 ± 6.62 months postoperatively) revealed that the rate of PCOA enlargement in 2 to 25.64 ± 6.62 months of follow-up was similar to that in 6 to 12 months of follow-up. Age was often a factor because of the increased risk of capsular-related complications following cataract surgery with intraocular lens implantation in children younger than two years (27). We enrolled 8 eyes of 7 patients younger than two years of age in this study. The results showed that the PCOA growth in patients younger than two years of age was similar to that in patients older than two years. Also, we performed correlation analyses between the change of PCOA or lens reproliferation area and factors such as age at the time of surgery, axial length growth, and follow-up duration. The results showed that the enlargement of PCOA did not significantly correlate with age at surgery, but it had a positive correlation with the growth of axial length from six months to last visit and follow-up duration. These results suggest that ocular development may have an impact on the changes in capsular outcomes, possibly due to a reduction in the mechanical force of the capsule after the loss of both anterior and posterior capsular components. The zonular fibers attached to the equatorial surface of the lens exert an outward pull on the lens (17). After lens aspiration, the capsule grows marginally (28), but other ocular components, such as corneal radii, develop significantly between 3 months and 6.5 years of age (29). Therefore, ocular development may enhance the centrifugal force through healthy zonular fibers, resulting in PCOA enlargement. As the age of patients in this study was limited to 0.5–6.67 years, a larger population and enrollment of a wider age range are needed to verify this finding.

IOL material and design may influence capsular outcomes (30). During this study, several different types of IOL were implanted, including SA60AT, Tecnis ZCB00, and A1-UV. However, these IOL materials are all hydrophobic acrylic, which makes up for the inconsistency of intraocular lens to some extent. Nevertheless, no significant anterior capsular contraction occurred in the eyes of our patients, and the implanted IOLs seemed safe.

For better intuitiveness, areas were converted to corresponding diameters for better guidance during surgery. The PCOD enlarged at a rate of 0.05 mm/month during the first six months after surgery; then, it enlarged at a slower rate of 0.01 mm/month. Accordingly, the PCOD is estimated to enlarge by approximately 0.5 mm in the first two years after surgery and may enlarge continuously thereafter, although a longer follow-up is required to confirm it. Therefore, it is reasonable to expect that the PCOD may enlarge 0.5- to 1.0-mm postoperatively. Generally, a 3.0- to 5.0-mm diameter of the posterior capsulorhexis is acceptable during pediatric cataract surgery (8, 31). Considering an expectation of 1.0 mm PCOD enlargement and a 0.5- to 1.0-mm margin necessary for IOL, a 3.0- to 4.0mm PCOD is more recommended for good long-term prognoses of in-the-bag IOLs according to the results of this study. It’s worth noting that some alternate approaches that can also prevent the incidence of VAO effectively, like optic capture through the posterior capsulorhexis and bag-in-the-lens without anterior vitrectomy, are also gaining more and more popularity (6, 32, 33). These surgical procedures have different requirements for the size of posterior capsulotomy. In the technique of “bag-in-the-lens”, manual anterior and posterior capsulorhexis of the same size (4.5 mm) is usually recommended (7). And another surgical procedure “posterior optic capture” suggested that the diameter of posterior capsulorhexis should be 1.0–1.5mm smaller than the IOL optic or 4.0- to 4.5mm (5, 6, 34).

At the last postoperative visit, lens reproliferation was acceptable in all eyes. In pediatric cataracts, LEC proliferation decreases with age (35). VAO induced by lens reproliferation most often occurs within one year after pediatric cataract surgery with primary IOL implantation (36). Our results showed that lens reproliferation in the first six months postoperatively had a significant correlation with age at the time of surgery. After six months, the lens reproliferation did not increase significantly. The transparency of the visual axis during this study was highly encouraging. However, longer follow-up is still needed to observe the occurrence of VAO.

This research studied the changes in capsular outcomes 1 to 2 years after pediatric cataract surgery with primary IOL implantation and demonstrated the PCOA expansion after the surgery. One limitation of this research was its relatively short follow-up period. We aim to follow up with these patients for a more extended period. Another limitation was the lack of ACO measurements. Indicators such as cornea diameters need to be further evaluated to understand the relationship between ocular development and changes in capsular outcomes.

In conclusion, the PCOA enlarged after surgery, and the enlargement was positively correlated with the follow-up duration and axial length growth. The anterior capsulorhexis diameter of 5.0–5.5 mm used in our study was safe and suitable for pediatric cataract surgery with IOL implantation, with a low incidence of severe contraction of the capsular opening and VAO. The posterior capsulorhexis or vitrectorhexis size should be considered to ensure good long-term prognoses. We recommend a posterior capsulorhexis or vitrectorhexis diameter of 3.0–4.0 mm for good visual axis transparency and protection of in-the-bag IOL.

## Data Availability Statement

The original contributions presented in the study are included in the article/supplementary material, further inquiries can be directed to the corresponding author.

## Ethics Statement

The research protocol was approved by the Institutional Review Board and Ethics Committee of Wenzhou Medical University, and was conducted according to the tenets of the Declaration of Helsinki. Informed consent was obtained from the parents of all participating children. Written informed consent to participate in this study was provided by the participants’ legal guardian/next of kin.

## Author Contributions

YF drafted the manuscript and collected patient information. DW and XD analyzed and interpreted the patient data. PC and YZ collected data. MH and ZL edited the patient photos. Y-eZ critically revised the manuscript for intellectual content and supervised the project. All authors read and approved the final manuscript.

## Conflict of Interest

The authors declare that the research was conducted in the absence of any commercial or financial relationships that could be construed as a potential conflict of interest.

## Publisher’s Note

All claims expressed in this article are solely those of the authors and do not necessarily represent those of their affiliated organizations, or those of the publisher, the editors and the reviewers. Any product that may be evaluated in this article, or claim that may be made by its manufacturer, is not guaranteed or endorsed by the publisher.
